# Chromatin Protamination and Catsper Expression in Spermatozoa Predict Clinical Outcomes after Assisted Reproduction Programs

**DOI:** 10.1038/s41598-017-15351-3

**Published:** 2017-11-09

**Authors:** S. Marchiani, L. Tamburrino, F. Benini, L. Fanfani, R. Dolce, G. Rastrelli, M. Maggi, S. Pellegrini, E. Baldi

**Affiliations:** 1 Dept. of Experimental and Clinical Medicine, Center of Excellence DeNothe, University of Florence, Florence, Italy; 2 Centro Procreazione Assistita “Demetra”, Florence, Italy; 3 Dept. of Experimental and Clinical Biomedical Sciences “Mario Serio”, Center of Excellence DeNothe, University of Florence, Florence, Italy

## Abstract

Identification of parameters predicting assisted reproductive technologies (ARTs) success is a major goal of research in reproduction. Quality of gametes is essential to achieve good quality embryos and increase the success of ARTs. We evaluated two sperm parameters, chromatin maturity and expression of the sperm specific calcium channel CATSPER, in relation to ART outcomes in 206 couples undergoing ARTs. Chromatin maturity was evaluated by Chromomycin A3 (CMA3) for protamination and Aniline Blue (AB) for histone persistence and CATSPER expression by a flow cytometric method. CMA3 positivity and CATSPER expression significantly predicted the attainment of good quality embryos with an OR of 6.6 and 14.3 respectively, whereas AB staining was correlated with fertilization rate. In the subgroup of couples with women ≤35 years, CATSPER also predicted achievement of clinical pregnancy (OR = 4.4). Including CMA3, CATSPER and other parameters affecting ART outcomes (female age, female factor and number of MII oocytes), a model that resulted able to predict good embryo quality with high accuracy was developed. CMA3 staining and CATSPER expression may be considered two applicable tools to predict ART success and useful for couple counseling. This is the first study demonstrating a role of CATSPER expression in embryo development after ARTs programs.

## Introduction

Infertility is a condition of global proportion affecting about 15% of couples^1^, expected to increase in the future. Assisted reproduction technologies (ARTs) are a valid and widely used treatment option for couple infertility. Although huge improvements in outcomes of ARTs have been made in the last few years, the successful pregnancy rate remains quite low, averaging, in European countries, 29.6% for *in vitro* fertilization (IVF) and 27.8% for intracytoplasmic sperm injection (ICSI)^2^. Lack of ART success implicates financial burden on both health services and patients and impacts negatively on life quality of couples. Failures in ART can be attributed to embryonic factors, as embryo quality plays a crucial role in the attainment of pregnancy^3^. Currently, no reliable markers are available to predict embryo quality and other early ART outcomes, which are related directly to the quality of the couple gametes. At present, male gamete assessment is based on semen analysis which is poorly predictive of both natural^4^ and assisted^5,6^ reproduction, as the semen of about 20–30% of normozoospermic men has low fertilizing ability^7^. Indeed, beside normal motility and morphology, a spermatozoon must have intact DNA and other essential features to be able to fertilize the oocyte and to allow a correct embryo development. Since identification/assessment of molecular markers of oocyte quality is basically unfeasible, the identification of sperm markers able to predict ART outcomes represents a priority to reduce negative psychological and economic consequences to the couples. In the present study, we focused on chromatin maturity status and expression of the calcium channel CATSPER, two essential features for correct sperm function.

During spermatogenesis histones are replaced by protamines to stabilize chromatin structure^8^. Such process allows organizing sperm DNA into a tightly packed structure to preserve paternal genome during the transit in male and female genital tracts until the interaction with the oocyte. Although about 15% of histones are physiologically retained by spermatozoa, a greater persistence of histones or a decreased protamination are an index of chromatin immaturity that can affect sperm quality and the fertilizing capacity^9^. Among the tests used to evaluate sperm chromatin compaction, Chromomycin A3 (CMA3) and Aniline Blue (AB) stainings are two simple, low cost and easy to perform methods. CMA3 competes with protamines for binding to DNA minor groove representing an indirect measure of protamination state. AB staining assesses the histone persistence by binding to lysine residues of these nuclear proteins. Although several studies^10,11^ evaluated the impact of chromatin compaction on ARTs, the heterogeneity of the ART outcomes taken into account and the lack of consideration of possible confounding factors such as female age and female infertility factors, do not allow to draw clear conclusions about the predictive ability of this parameter. Indeed, it is known that female age is an important predictor of ART success, in particular, the probability of pregnancy decreases markedly after the age of 35 years^12,13^. Similarly, some female infertility causes (i.e. poor ovarian reserve, endometriosis and polycystic ovary syndrome) are associated with reduced chance of fertilization, implantation and pregnancy in ARTs^14–16^. Another key limiting factor in female fertility is oocyte maturation, determined by the acquisition of a series of competencies during follicular development which allow reaching the metaphase II (MII) stage^17^.

Studies on animal models highlighted the key role of the sperm-specific calcium channel CATSPER (Cationic Channel of Sperm) in the development of hyperactivated motility^18–20^, an essential sperm characteristic. The functional channel is formed by four homologous subunits (CATSPER 1–4) and at least three auxiliary subunits^21,22^. In the mouse, Qi *et al*.^19^ demonstrated that lack of any CATSPER subunits leads to complete absence of the channel in mature spermatozoa. Recently, we demonstrated a positive correlation between the level of expression of CATSPER1 subunit (measured by a flow cytometric method^23,24^) and sperm number, progressive motility and hyperactivation^24^, suggesting that expression of the channel may be indicative of sperm quality. On the other hand, the few men with deletions in CATSPER subunits genes, leading to absence of a functional channel in spermatozoa, are infertile and show poor semen quality^25,26^. However, until now, it is unknown whether CATSPER expression is implicated in human fertilization process or related to ART outcomes.

We here assessed chromatin maturity status (by CMA3 and AB staining) and CATSPER1 expression (by a flow cytometric method) in semen samples from male partners of 206 couples undergoing ART treatments. To determine if these male molecular markers may be predictive of ART success, it was evaluated their association with ART outcomes, both as single test and in combination, taking into account several confounding factors affecting the statistical analysis.

## Results

### Chromatin compaction and ART outcomes

**Figure 1:**
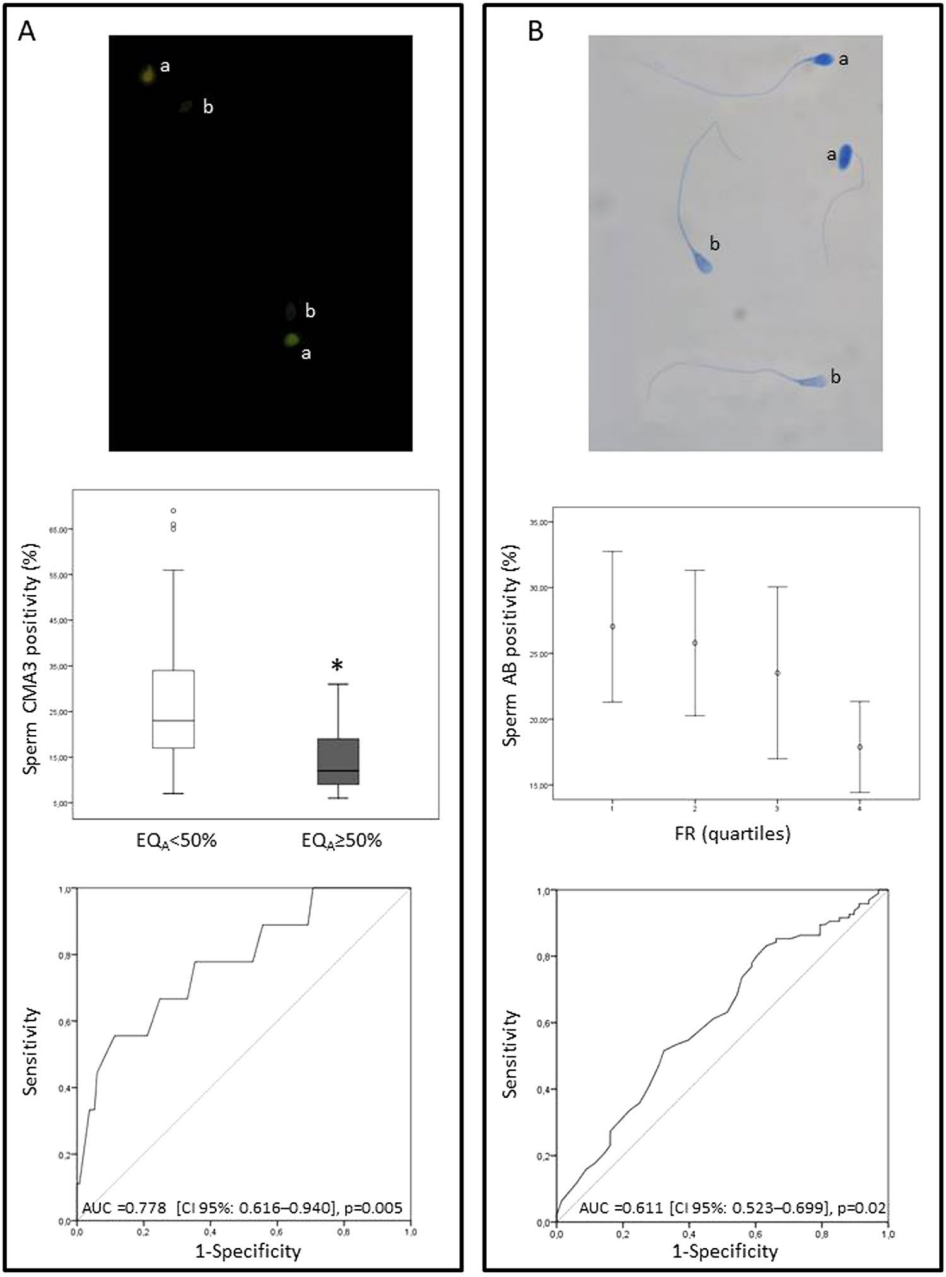
Association between CMA3 or AB and ART outcomes. (**A**) upper panel: Examples of spermatozoa positive (a) or not (b) after staining with CMA3; middle panel: Box plot representing sperm CMA3 positivity in the two groups with embryos with A quality (EQ_A_) <50% (n = 133) and ≥50% (n = 9); lower panel: Receiver Operating Characteristic curve of the ability of CMA3 positivity to predict the attainment of at least 50% EQ_A_. (**B**) upper panel: Examples of spermatozoa positive (a) or not (b) after staining with AB; middle panel: Association between sperm AB positivity and FR. Note that statistical analyses have been performed using FR as a continuous variable, even if grouped in quartiles for graphical purposes; lower panel: Receiver Operating Characteristic curve of the ability of AB positivity to predict the attainment of at least 80% FR. *p ≤ 0.05; FR: Fertilization rate; EQ_A_: embryos with A quality; AUC: Area under the curve; CI: confidence interval.

Age and semen parameters values of the male partners of the 206 couples included in the study are shown in Table 1. None of the semen parameters evaluated on the day of pick up nor male age were related to early ART outcomes, pregnancy achievement or delivery (not shown). In addition, semen parameters on the day of pick up were similar in groups with EQ_A_ <50% or ≥50%, FR <80% or ≥80% and in couples achieving or not clinical pregnancy or ending or not with delivery (Table 1), confirming lack or poor predictivity of ART outcomes by semen parameters. The median percentages of spermatozoa showing chromatin immaturity revealed by AB (n = 163) and CMA3 (n = 149) techniques were 20.0% [13.0–28.0] and 23.0% [16.0–33.5], respectively. The two measures were significantly correlated (r = 0.5, p < 0.0001, n = 147). Correlations between levels of CMA3 and AB staining and ART outcomes are reported in Table 2. The percentage of CMA3 positive spermatozoa resulted negatively associated with EQ_A_ (Table 2) even after adjusting for female age, female factor and number of MII oocytes (adj. β = −0.2, p = 0.04). No significant correlations were found between CMA3 and other ART outcomes. After categorizing couples according to the percentage of embryos with A quality (EQ_A_ ≥50% and EQ_A_ <50%), CMA3 positivity was significantly lower when embryo quality was higher (EQ_A_ <50%: 23.0 [16.5–34.5], n = 133; EQ_A_ ≥50%: 12.0 [8.5–22.0], n = 9, p = 0.005, Fig. 1A, middle panel). The difference was confirmed in a confounder-adjusted model (p = 0.02). To establish a CMA3 value able to predict an EQ_A_ ≥50%, ROC analysis was performed (Fig. 1A, lower panel). At a threshold of 19.5%, CMA3 predicted the attainment of EQ_A_ ≥50% with 78% sensitivity and 65% specificity. Applying a logistic regression model, we found that the probability of obtaining EQ_A_ ≥50% was higher when the CMA3 positivity was ≤19.5% (OR = 6.6, CI 95%: 1.29–33.63, p = 0.02).

**Table 1:** Median [IQR range] values of male age and semen parameters in male partners of infertile couples included in our study.

	Entire cohort (n = 206)	Women age			FR			EQ_A_			Pregnancy			Delivery		
		≤35(n = 115)	>35 (n = 91)	p	<80% (n = 89)	≥80% (n = 117)	p	<50% (n = 181)	≥50% (n = 17)	p	Yes (n = 56)	No (n = 120)	p	Yes (n = 42)	No (n = 14)	p
Male age (years)	38.0 [35.0–41.0]	36.0 [34.0–39.0]	39.0 [37.0–42.0]	p < 0.001	38.0 [35.0–41.0]	37.0 [35.0–41.0]	ns	38.0 [35.0–41.0]	37.0 [35.5–40.5]	ns	37.0 [34.3–40.8]	38.0 [34.5–41.0]	ns	37.0 [34.5–40.5]	37.5 [34.8–41.3]	ns
Semen volume (ml)	3.0 [2.0–4.0]	3.0 [2.0–4.0]	3.0 [2.0–4.0]	ns	3.0 [2.0–4.0]	3.0 [2.0–4.0]	ns	3.0 [2.0–4.0]	4.0 [2.5–4.0]	ns	3.0 [2.0–4.0]	3.0 [2.0–4.0]	ns	3.0 [2.0–4.0]	2.0 [1.2–5.0]	ns
Sperm concentration (x10^6^/ml)	39.5 [20.0–70.0]	36.0 [18.0–66.0]	44.0 [22.0–78.0]	ns	37.0 [20.0–79.0]	40.0 [20.0–62.5]	ns	40.0 [19.5–70.0]	50.0 [21.0–75.0]	ns	37.0 [18.3–70.0]	38.5 [20.0–70.8]	ns	38.0 [17.5–71.5]	36.5 [19.0–61.0]	ns
Total number of spermatozoa (x10^6^/ejaculate)	101.0 [60.0–200.0]	90.0 [51.0–200.0]	110.0 [65.0–210.0]	ns	96.0 [55.3–198.5]	103.8 [65.3–219.0]	ns	100.0 [54.0–200.0]	120.0 [77.0–228.0]	ns	80.0 [48.6–220.0]	94.0 [73.0–280.0]	ns	84.5 [53.0–227.3]	72.0 [38.0–180.0]	ns
Progressive motility (%)	45.0 [34.8–57.0]	46.0 [35.0–58.0]	45.0 [33.0–55.0]	ns	43.0 [30.5–55.0]	48.0 [36.5–58.5]	ns	45.0 [34.5–56.0]	50.0 [31.0–59.5]	ns	47.5 [35.0–60.0]	43.5 [33.8–55.0]	ns	48.0 [35.5–60.0]	38.5 [25.0–57.0]	ns
Normal morphology (%)	11.0 [4.0–11.0] (n = 106)	11.0 [3.3–11.0] (n = 64)	11.0 [4.0–11.0] (n = 42)	ns	10.0 [1.0–11.0] (n = 42)	11.0 [5.0–11.0] (n = 64)	ns	10.5 [4.0–11.0] (n = 98)	11.0 [3.5–11.0] (n = 4)	ns	10.5 [2.5–11.0] (n = 26)	9.0 [3.0–11.0] (n = 63)	ns	11.0 [5.0–11.0] (n = 22)	10.0 [1.0–11.0] (n = 6)	ns

Semen parameters were evaluated on the day of insemination in all subjects except morphology which was evaluated in 106 subjects. p: probability; ns: not significant; FR: fertilization rate; EQ_A_: embryos with A quality.

**Table 2:** Correlations between sperm AB or CMA3 positivity and ART outcomes (FR, CR, EQ_A_, IR, PR and DR) in the total cohort of couples.

ART outcome	Total cohort	
	CMA3 (%)	AB (%)
FR	r = −0.05, p = 0.6, n = 149	**r = −0.2, p = 0.004, n = 163**
CR	r = −0.01, p = 0.9, n = 145	r = −0.02, p = 0.7, n = 159
EQ_A_	**r = −0.2, p = 0.03, n = 142**	r = −0.02, p = 0.8, n = 156
IR	r = 0.01, p = 0.9, n = 124	r = 0.02, p = 0.8, n = 138
PR	r = 0.03, p = 0.7, n = 124	r = 0.1, p = 0.4, n = 138
DR	r = −0.04, p = 0.4, n = 40	r = 0.2, p = 0.2, n = 45

FR: Fertilization rate; CR: Cleavage rate; EQ_A_: embryos with A quality; IR: Implantation rate; PR: Pregnancy rate; DR: Delivery rate.

r: Spearman’s correlation coefficient, p: probability, n: number of couples.

AB positivity was negatively associated with FR (Table 2 and Fig. 1B, middle panel), even in a confounder-adjusted model (adj. β = −0.2, p = 0.02). No correlation was observed with other ART outcomes (Table 2). To determine the accuracy of AB in predicting the FR, we used ROC as a binary classifier system choosing a value of 80% FR, which corresponds to median value of the cohort (Fig. 1B, lower panel). At a threshold of 25.5%, AB predicted FR ≥80% with a good sensitivity (78%) but low specificity (41%) (Fig. 1B, lower panel). A post hoc binary logistic regression analysis indicated that the probability of obtaining an FR ≥80% was higher when the AB positivity was ≤25.5%, with an OR of 2.3 (CI 95%: 1.19–4.79, p = 0.01).

To further investigate whether female age affects the association between CMA3 and AB positivity and ART outcomes, a subgroup analysis was performed according to women age ≤35 or >35 years (the median age of our cohort and the threshold above which the risk of miscarriage and chromosomal aberrations significantly increase^27,28^ and the probability of pregnancy decreases^12,13^). In couples with women ≤35 years (n = 115), the subgroup analysis confirmed the significant difference in CMA3 positivity between EQ_A_ <50% and EQ_A_ ≥50% (not shown) as well as the correlation between AB positivity and FR (not shown) found in the entire cohort. In addition, the OR to predict the achievement of an EQ_A_ ≥50% for CMA3 threshold of 19.5 increased to 10.7 (CI 95%: 1.78–97.74, p = 0.04), and the OR to obtain an FR ≥80% for the AB threshold of 25.5% to 2.8 (CI 95%: 1.12–6.95, p = 0.03).

### CATSPER1 expression and ART outcomes

**Figure 2:**
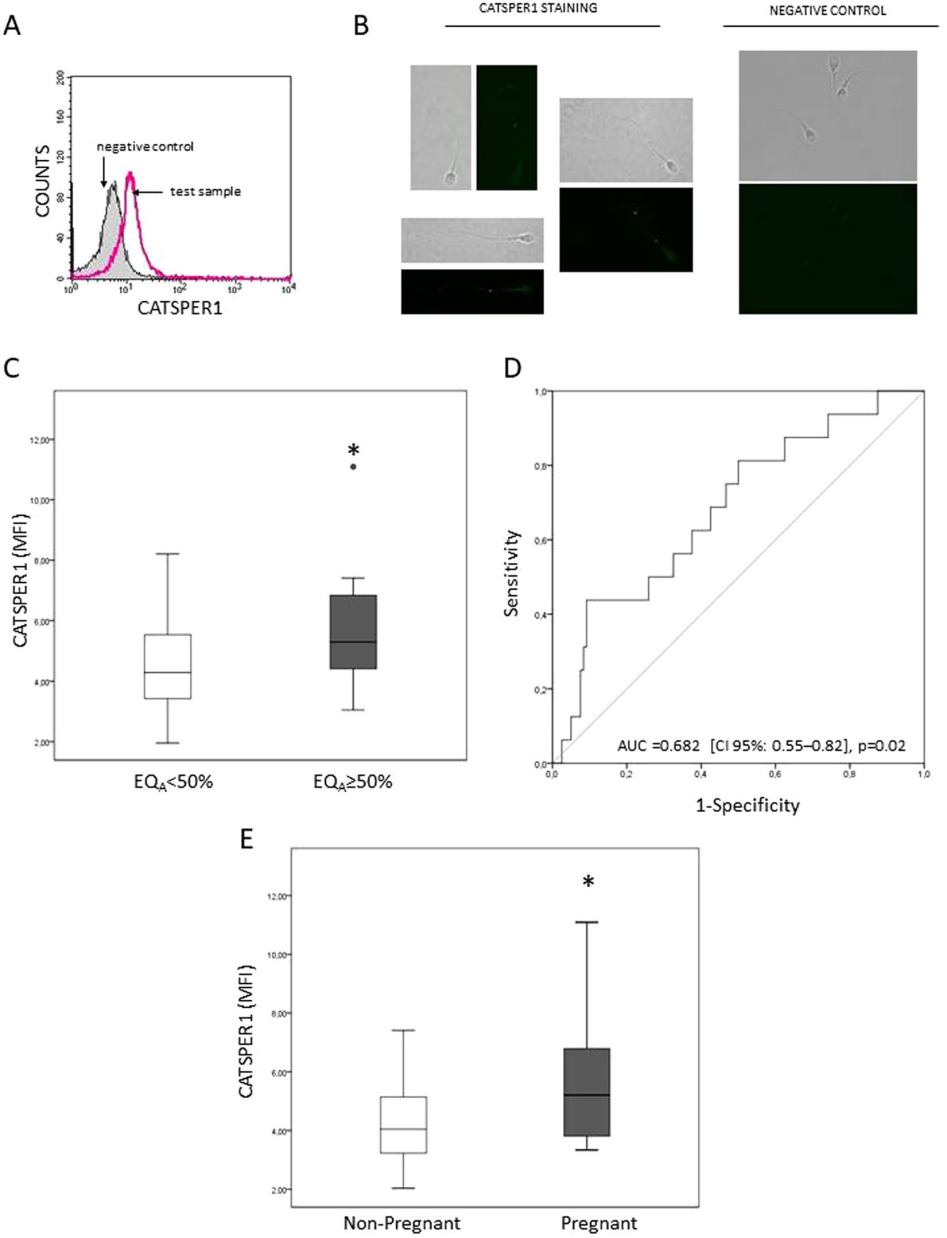
Association between CATSPER1 expression and embryo quality and pregnancy achievement. (**A**) Typical histogram of CATSPER1 fluorescence in spermatozoa showing overlay of test sample (open) and corresponding negative control (rabbit serum, grey). (**B**) Images obtained by fluorescence microscopy and relative bright field of spermatozoa stained with CATSPER1 antibody (left) or rabbit serum (right). (**C**) Box plot representing CATSPER1 expression in groups with embryo quality A (EQ_A_) <50% (n = 120) and ≥50% (n = 16). (**D**) Receiver operating characteristic curve of the ability of CATSPER1 MFI to predict the attainment of at least 50% EQ_A_. E: Box plot representing CATSPER1 expression in couples achieving (n = 20) or not (n = 45) clinical pregnancy in the subpopulation of couples with female partners ≤35 years. *p ≤ 0.05; AUC: Area under the curve; CI: confidence interval; MFI: median fluorescence intensity.

In the 141 male partners of the cohort, the median value of CATSPER1 expression was 4.5 [3.5–5.8]. At a first glance, CATSPER1 expression was found to be correlated with no ART outcome (not shown). However, after adjustment for female age, female factor and number of MII oocytes, a positive correlation between CATSPER1 expression and EQ_A_ was unmasked (adj. β = 0.2, p = 0.03). Figure 2B shows that CATSPER1 MFI was significantly higher in the group with EQ_A_ ≥50% (5.3 [4.3–6.9, n = 16] in EQ_A_ ≥50% vs 4.3 [3.4–5.6, n = 120] in EQ_A_ <50%, p = 0.002). The difference was also confirmed after adjusting for confounders (p = 0.03). A ROC curve analysis was performed to determine the threshold of CATSPER1 expression associated with EQ_A_ ≥50% (Fig. 2C). We found that the attainment of a good embryo quality was predicted with a specificity of 91% and a sensitivity of 44% at the CATSPER1 value of 6.74. By binary logistic regression, we found that above this threshold (CATSPER1 ≥6.74) the OR to obtain an EQ_A_ ≥50% was 14.3 (CI 95%: 3.50–58.09; p < 0.0001).

In the subgroup of younger women (≤35 years), CATSPER1 expression was significantly correlated with EQ_A_, IR and PR (Table 3), even after adjusting for female factor and number of MII oocytes (EQ_A_: adj. β = 0.3, p = 0.005; IR: adj. β = 0.3, p = 0.03; PR: adj. β = 0.2, p = 0.05). As in the entire cohort, CATSPER1 MFI was higher in couples with EQ_A_ ≥50% (6.8 [4.5–7.2, n = 9] vs 4.1 [3.4–5.3, n = 64] in EQ_A_ <50%, p = 0.006), even in a confounder-adjusted model, p = 0.02. In addition, in this subgroup, CATSPER1 expression was higher in couples achieving clinical pregnancy (5.2 [3.7–6.8, n = 20] vs 4.05 [3.2–5.1, n = 45] in the non-pregnant, p = 0.02; Fig. 2D). This difference was confirmed after adjusting for female factor and number of MII oocytes (p = 0.05). CATSPER expression was also slightly, but not significantly, higher in couples ending with a delivery (median values: 5.9 [3.7–6.9, n = 13] vs 4.6 [3.6–6.4, n = 7], p = ns). Logistic regression analysis showed that when CATSPER1 MFI was ≥6.74 (see above) the odds to obtain EQ_A_ ≥50% (OR = 17.7, CI 95%: 3.07–102.07, p = 0.001) and to achieve clinical pregnancy (OR = 4.4, CI 95%: 1.08–18.21, p = 0.04) were higher.

**Table 3:** Correlations between CATSPER1 expression and ART outcomes (FR, CR, EQ_A_, IR, PR and DR) in the subpopulation of couples with female partners ≤ 35 years.

ART outcome	Female age ≤ 35 years
	CATSPER1 (MFI)
FR	r = −0.1, p = 0.2, n = 74
CR	r = 0.05, p = 0.7, n = 74
EQ_A_	**r = 0.2, p = 0.05, n = 73**
IR	**r = 0.3, p = 0.02, n = 63**
PR	**r = 0.3, p = 0.01, n = 63**
DR	r = 0.14, p = 0.6, n = 19

FR: Fertilization rate; CR: Cleavage rate; EQ_A_: embryos with A quality; IR: Implantation rate; PR: Pregnancy rate; DR: Delivery rate.

r: Spearman’s correlation coefficient, p: probability, n: number of couples.

### Development of an Embryo quality prediction model

**Figure 3:**
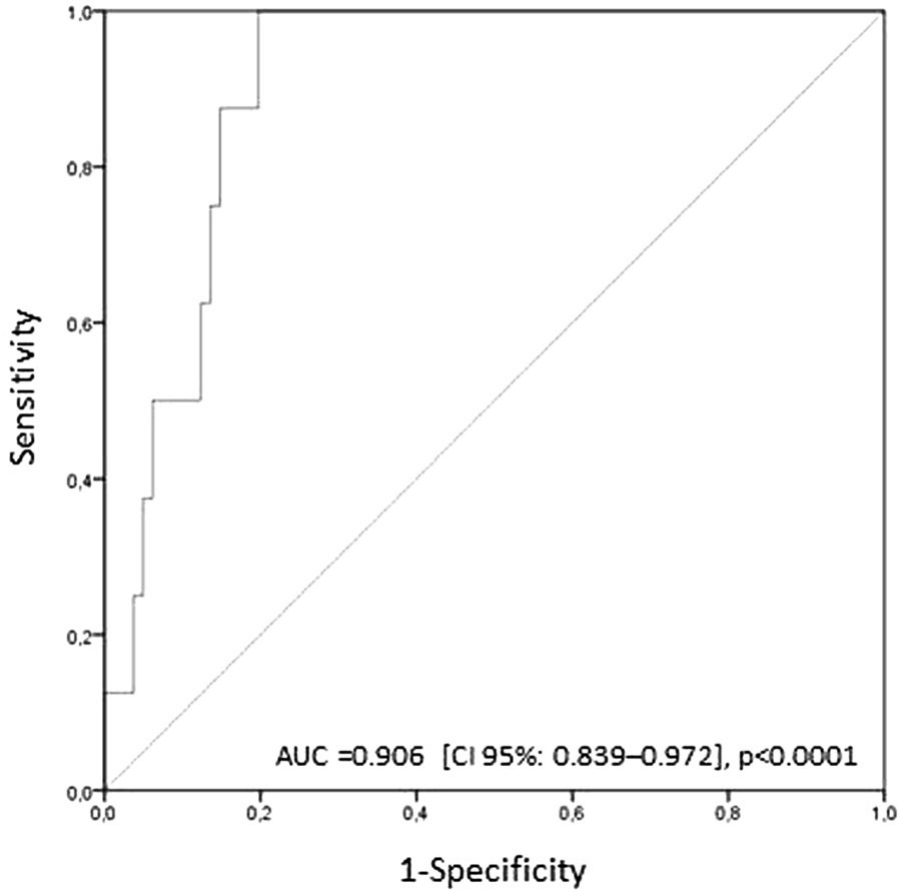
Predictive model of embryo quality. Receiver operating characteristic curve of the probability of obtaining a percentage of embryo quality of type A ≥50% as predicted by the proposed binary logistic regression model described in the text. AUC: area under the ROC curve; CI: confidence interval.

The results concerning the ability of both CMA3 and CATSPER1 levels to predict EQ_A_ ≥50% prompted us to build up an embryo quality prediction model. As mentioned above, embryo quality is considered, indeed, as a strong predictor of implantation, pregnancy and live birth after ARTs^3,13,29,30^. In particular, in our cohort, couples with an EQ_A_ ≥50% had 3.17 higher probability of pregnancy (CI 95%: 1.04–9.62, p = 0.04). Considering previously published studies regarding the clinical significance of female parameters on ART outcomes, the model not only included CMA3 positivity and CATSPER1 expression, but also female age^12,13^, female factor^14–16^ and number of MII oocytes^17^. Table 4 reports coefficients from the logistic regression model that could be used to calculate a probability of obtaining an EQ_A_ ≥50% for all couples. The equation describing the probability of developing good embryos is:

$${\rm{P}}={e}^{x}\div(1+{e}^{x})$$

with x = b0 + b1*p1 + b2*p2 + b3*p3 + b4*p4+b5*p5, where p1…p5 are our predictors’ values and b0…b5 are the coefficients derived from the model (Table 4). We assessed the discrimination of the predictive model by calculating the area under the ROC curve (Fig. 3), which denotes that our model is able to predict the achievement of an EQ_A_ ≥50% with good probability. The goodness of fit of the model was evaluated using the Hosmer–Lemeshow statistic test. Such test demonstrated no statistically significant difference between the predicted and observed values (x² = 3.107, p = 0.927).

**Table 4:** Binary logistic regression model to predict embryo quality type A.

Covariates	Coefficient	SE	p-value	CI 95%	
CMA3 (%)	b1:–0.178	0.070	0.012	0.730	0.961
CATSPER1 (MFI)	b2:0.971	0.357	0.007	1.311	5.318
Number of MII oocytes	b3:0.174	0.131	0.183	0.921	1.537
Female age	b4:–0.002	0.197	0.991	0.681	1.474
Female factor	b5:–1.536	1.297	0.236	0.017	2.734
Constant	b0:–5.458	7.139	0.444		

SE: standard error; CI: confidence interval; P-value probability.

## Discussion

Despite the considerable progresses done in the latest years, the rate of clinical pregnancy after ARTs remains low (about 30%). The identification of one or more parameters able to predict the outcomes of ARTs appears mandatory to increase the percentage of success, avoid psychological stress, optimize the correct counselling of the couples and reduce the costs. Among the critical steps for ART success, the development of a good quality embryo appears of upmost importance, as it is highly related to the attainment of clinical pregnancy, as demonstrated in the current and previous studies^3^. We show here that two sperm parameters, chromatin compaction as evaluated by CMA3 staining and expression of the sperm specific calcium channel subunit CATSPER1, show significant correlations with development of good quality embryos, suggesting a certain grade of dependence of embryo quality from the two parameters. Based on the evaluation of the two sperm parameters, and including other parameters that are known to affect embryo quality, we developed a model which resulted able to predict the ability of couples to obtain good quality embryos with high accuracy. In addition, we demonstrated that expression of CATSPER1 unveils the importance of sperm quality in pregnancy achievement in couples with female age below 35 years.

In our study, chromatin maturity status has been assessed by two methods, CMA3 and AB, which are widely described in the literature^10,11^. Theoretically the two methods should evaluate the same sperm aspect and, indeed, a positive relationship is present between the results obtained with the two techniques (31–32 and present study). However, the correlation is not as tight as expected (r = 0.5 in the present study and 0.4 in the Iranpour *et al*.^31^ study), suggesting that histones retention (evaluated by AB) does not necessarily correspond to a decrease of protamination (evaluated by CMA3) and vice versa. Such conclusion is strengthened by the demonstration that CMA3 and AB results are differently related to ART outcomes (present study and refs^31,33^). In particular, we found that whereas CMA3 values are associated to the development of good quality embryos, AB staining is only weakly associated with fertilization rate.

Our data on the association between CMA3 positivity and embryo quality is in apparent contrast with two previous studies, where such correlation was not observed^34,35^. Of note, the latter studies were conducted on a small number of couples and without considering female factor and/or female age^34^ or simply excluding female factors from the analysis^35^. We now show not only that CMA3 predicts achievement of EQ_A_ ≥50% in the entire cohort, but also that the OR for this prediction is almost twice higher in couples with younger women. This suggests that women age could mask the sperm contribution to embryo development and evidences the necessity of considering female age and female factors in the analysis on the impact of sperm characteristics on ARTs results. Protamines are important actors in the fertilization process: they are exchanged for maternal nucleosomes shortly after fertilization^36^ and this process may be important for reprogramming to totipotency of the zygote^37^.

At difference with other studies^31–33,38,39^ we did not observe any relationship between CMA3 and fertilization rate, probably because of the high FR obtained in our study (about 80%). However, a negative relationship with FR was observed for AB staining, suggesting that the chromatin status may impact the ability of spermatozoa to fertilize the oocyte. It appears from our study that histones persistence, rather than protamination, is more important in the fertilization process. Other groups did not find any associations between sperm AB staining and ART outcomes probably due to the small number of included couples^31,32,40^. Further studies are needed to better understand the role of histone persistence in fertilization.

Overall our results remark the importance of chromatin maturation for ART success. On the other hand, an incorrect chromatin compaction exposes spermatozoa to DNA damage^41^ which can impact ART outcomes^42–44^. With regard to most assays assessing sperm DNA fragmentation, the tests detecting chromatin immaturity used in our study are easy and rapid to perform, require a low number of cells and no technologically advanced instruments, hence they could be performed in any ART centre. All these advantages make CMA3 and AB methods valuable tools to support routine semen analysis in the diagnosis of male partner of infertile couples.

The present study is the first one evaluating the association between CATSPER1 protein expression (likely reflecting the expression of the entire CATSPER channel^19^) and ART outcomes. A role of CATSPER channel in sperm-oocyte interaction is expected since KO mice for any CATSPER subunit are infertile^19^ because their spermatozoa are unable to develop hyperactivated motility and to penetrate the zona pellucida. In addition, men with mutations/deletions in genes encoding for CATSPER subunits show fertility problems^26^ likely due to absence of a functional channel^25^. In human spermatozoa CATSPER is activated with a non-genomic mechanism^45^ by progesterone^46,47^, a hormone present in high concentrations at fertilization site, by a rise of intracellular pH, such as induced by 4-aminopyridine^48^, and by other components present in follicular fluid^49^. Sperm responsiveness to both progesterone and 4-aminopyridine are correlated to fertilization rate in ART programs^48,50,51^. We found here that CATSPER1 expression levels are higher in couples with good embryo quality in the entire cohort and in couples achieving clinical pregnancy in the subgroup of young women. Most importantly, we demonstrate that, at the threshold of ≥6.74 MFI, CATSPER1 expression predicts development of good embryos with an OR of 14.3. This result indicates that a higher expression of the channel in sperm is important for a correct human embryo development. However, whether CATSPER channel is indicative of a sperm characteristic implicated in embryo development or is required itself for the process is presently unknown. In a previous paper by our group we demonstrated that the percentage of human spermatozoa expressing CATSPER1 predicts with high accuracy the sperm ability to hyperactivate^24^, suggesting that the channel is involved in the capacitation process leading to hyperactivation *in vitro*. It has been demonstrated that spermatozoa of CATSPER KO mice partially reacquire the ability to fertilize the oocyte by *in vitro* fertilization and to develop blastocysts when capacitation is accelerated by the addition of the calcium ionophore A23187, evidencing a crucial role of CATSPER in capacitation^52^. Recently, in bovine, it has been demonstrated that artificial induction of capacitation is important for blastocyst formation also when fertilization is obtained with ICSI^53^. Capacitation is essential for mammalian male fertility, as demonstrated by several KO models where the process is impaired^54,55^. Interestingly, Navarrete *et al*.^52^ showed that artificial induction of capacitation in some of these models (including, as mentioned above, CATSPER) restores the fertilizing ability of spermatozoa *in vitro*. We speculate that a higher CATSPER1 expression leads to a higher degree of capacitation of spermatozoa which is important for embryo development. Whereas the reason why lack of capacitation can affect fertilization competence in *in vitro* fertilization is understandable (lack of hyperactivated motility, inability to respond to acrosome reaction stimuli), why it might influence embryo development is obscure. Capacitated spermatozoa show many features that may affect oocyte activation and embryo development^56^, including elevated intracellular calcium levels^57^, tyrosine phosphorylation of proteins^58,59^, and modifications of the extracellular membrane composition^60^. Lack or low CATSPER expression may result in alteration of capacitation-related sperm calcium balance that could impact on oocyte activation. A recent study demonstrated that sperm expression of the protein PAWP is associated with embryo development in couples undergoing ICSI^61^ although, also in this case, the mechanisms involved in such action are poorly defined. Of note, a recent study demonstrated the importance of the sperm calcium channel TRP-3 in mediating the calcium wave occurring at fertilization in C. elegans oocyte^62^. To assess the role of CATSPER channel during fertilization and embryo development further studies are necessary in animal models.

The involvement of CATSPER1 expression in EQ is reinforced by our results within the younger women subgroup, where the same parameter was associated with and predicted pregnancy achievement with an OR of 4.4. The association between CATSPER1 and pregnancy achievement could simply reflect the higher embryo quality obtained in subjects with higher CATSPER1 expression. The relationship between CATSPER1 and pregnancy is observed in the subgroup with younger women, where the probability of pregnancy is higher and the contribution of male factor is likely unmasked.

None of the sperm parameters evaluated in our study were related to delivery rate. It should be considered that there are many factors that may influence a term pregnancy and the birth of a healthy child, some of which could be independent from the fact that clinical pregnancy has been obtained by ARTs.

Since both CMA3 and CATSPER1 are able to discriminate between low and high quality embryos, we introduced these markers in a model including also female parameters (female age, female factor and number of MII oocytes) in order to ameliorate the prediction value of EQ_A_. The probability of developing a good quality embryo, derived from such model, results more accurate than the single parameters. Moreover, in the present study, the predictive and observed values did not differ, confirming the reliability of the model. Several prediction models focusing on embryo quality, clinical pregnancy or live births (using IVF or ICSI) as primary outcome are present in the literature. Such studies include female factors (age, causes of infertility, hormone levels etc.) and/or general couple data (history and type of infertility) omitting entirely male parameters or simply considering the presence/absence of male factor infertility^63–66^. To our knowledge, this is the first study to present a novel model encompassing both female parameters and sperm intrinsic characteristics, such as the chromatin maturity status and expression of CATSPER1, as available prognostic factors in prediction models for IVF/ICSI outcomes.

This study has the strength of determining the impact of chromatin immaturity and CATSPER1 expression on ART outcomes taking into account female age, female factor and number of MII oocytes as confounders in the statistical analysis in a large number of couples. The study has some limitations. In particular, CATSPER1 expression was evaluated only in subjects with a sufficient number of spermatozoa to allow the determination (i.e. when it was possible to harvest 10 million spermatozoa from the entire ejaculate before selection for ARTs). Thus, patients with a low initial sperm number or severe male factor were not included. Since CATSPER1 expression is positively related with sperm number^24^, it is possible that subjects with low CATSPER1 expression were less represented in our cohort.

In conclusion, our study demonstrates that sperm histone retention plays a role in oocyte fertilization, whereas sperm protamine content and expression of CATSPER1 are involved in the development of good quality embryos. Combining the latter two markers with female age and female factor, we developed a prediction model of embryo quality which could be applicable in clinical practice and in the management of couples undergoing ARTs.

## Materials and Methods

### Study design and participants

The experimental protocol has been approved by the internal ethical committee of Demetra ART Center of Florence (Italy). We enrolled in a prospective cohort study 206 consecutive couples undergoing ART cycles at the Demetra ART Center of Florence (Italy) from March 2015 to October 2016. The obtainment of an informed written consent from the couples was the only criterion for inclusion in the study. All the couples were informed that, after the normal clinical practice for the ART treatment, the eventual remaining semen or selected spermatozoa would be used for the study.

The infertility diagnosis was: 56% female factor, 18% male factor, 10% male and female factor in combination and 16% unexplained. 156 couples were treated with ICSI and 50 with IVF. In 30 couples it was not possible to perform fresh transfer. Indications for deferred embryo transfer were: risk of ovarian hyperstimulation syndrome^67^, elevated progesterone levels (≥1.5 ng/ml) and inadequate endometrium on the trigger day^68^ in 20, 4 and 6 cases, respectively. To avoid a potential confounding bias due to different embryo transfer (fresh or frozen), implantation rate, pregnancy rate and delivery rate were calculated only for fresh embryo transfer (176/206). We transferred 1 embryo in 45 cases (26%), 2 in 124 (70%) and 3 in 7 (4%).

The median age of subjects was 35 [23–43] and 38 [27–55] years for female and male partners, respectively.

### Ovarian stimulation, IVF, ICSI, and Embryo Development

All the patients were treated according to the standard ovarian stimulation protocols of the clinic: 1) midluteal-phase GnRH-agonist (triptorelin, Decapeptyl, Ipsen Pharma) long protocol, followed by gonadotropin stimulation; 2) follicular phase GnRH-agonist/flare protocol (triptorelin, Decapeptyl, Ipsen Pharma), started with gonadotropin stimulation; 3) short protocol including gonadotropin stimulation from day 2 of the cycle, combined with a flexible antagonist protocol (cetrorelix 0.25 mg/day Cetrotide, Merck Serono or ganirelix 0.25 mg, Orgalutran, MSD Italia). In all cases follicle stimulation was performed with individual dosage of recombinant follicle stimulating hormone r–FSH (Gonal F, Merk Serono or Puregon, MSD Italia) or of highly purified human menopausal gonadotropin hMG (Meropur, Ferring), with a starting dose ranging from 150 to 450 IU, according to age, body mass index, ovarian reserve index and response to previous ovarian stimulation. The dose was then modified according to the ovarian response (determined by serum estradiol levels and ultrasound evaluation at 2 days interval), until at least two follicles reached 17 mm in mean diameter. Finally, oocytes maturation was induced by injection of 5000 IU of u-hCG (Gonasi, Ibsa Farmaceutici Italia) or 250 µg r-hCG (Ovitrelle, Merck Serono).

Gonadotropin stimulation and GnRH-agonist or GnRH-antagonist was continued until the day of hCG triggering, when progesterone levels were measured. Oocytes retrieval was performed about 35 hours later by sonographically guided puncture of the follicles, under sedation and local anesthesia.

For IVF, undecumulated oocytes were incubated overnight with about 50.000 spermatozoa/oocyte in Continuous Single Culture® Complete medium (Irvine Scientific, Santa Ana, CA, USA). For ICSI, Nikon Eclipse TE2000-S microscope equipped with Narishige IM-9B Microinjector was used. After 18 ± 1 hours from insemination (IVF) or after 17 ± 1 hours from microinjection (ICSI), oocytes were assessed for 2 pro-nuclei presence.

Continuous Single Culture® Complete medium was used for embryo culture. After 24 ± 1, 44 ± 1 and 68 ± 1 hours, pace of division, degree of fragmentation, size and symmetry of the blastomeres were evaluated by Nikon Eclipse TE2000-S microscope (Nikon, Tokyo, Japan). Embryos were incubated in a MINC benchtop incubator (Cook Medical, Bloomington, USA) at 37 °C, 6% CO_2_ and 5% O_2_ and were scored according to the criteria detailed in Supplementary Fig. 1. Embryos showing the best properties were classified into A class. Embryos showing slight deviation in the degree of fragmentation (5–30%), symmetry and division pace were classified into B and B/C (Supplemental Fig. 1). More considerable deviations were the cause for classifying them into C and D. Degenerated or arrested embryos (type E) were not transferred. Surplus transferable embryos were cryopreserved.

After 3 days post oocyte retrieval, embryos were transferred into the uterus.

Luteal support was given to all patients, administered as intravaginal micronized progesterone (Progeffik, 200 mg three times daily, EFFIK Italia), from the day after oocyte pick up until 12 days after embryo transfer, when serum hCG was measured. In case of positive hCG levels, clinical pregnancy was verified by ultrasound about 15 days later.

### Sperm preparation

Semen samples were collected by masturbation after 2–7 days of abstinence on the day of oocyte insemination. Sperm number, progressive motility and morphology were evaluated after liquefaction at 37 °C, according to WHO criteria^69^. Briefly, sperm number was evaluated by improved Neubauer chamber after appropriate dilution, motility by Nikon Eclipse TE2000 microscope scoring at least 100 spermatozoa/slide and morphology after Diff-Quick staining^69^. Sperm selection for oocyte insemination was performed by swim up (95 samples) or density gradient centrifugation (111 samples), according to sample characteristics. Swim up was performed by washing seminal fluid with Sperm Wash Medium (Irvine, Santa Ana, CA, USA) supplemented with 1% human serum albumin (HSA), and centrifuging at 300 g for 10 min. The obtained pellet was gently layered with 1 ml of the same medium and incubated at 37 °C. After 45 min, 800 µl of the upper medium phase was collected.

Density gradient centrifugation was performed layering 1 ml semen samples on 1 ml 45% and 90% stratified PureSperm (Nidacon, Gothenberg, Sweden) fractions (prepared in Sperm Wash Medium /HSA medium) and centrifuged at 300 g for 10 min at room temperature (RT). The resulting pellet was collected and transferred to separate test tubes. Then, each fraction was washed with 1 ml of Sperm Wash/HSA medium and then re-suspended in the same medium.

After selection, the obtained fraction was checked for sperm count and motility, kept at 37 °C in the same medium and used to inseminate the oocytes within 15 minutes from selection.

### Sperm chromatin immaturity

Sperm chromatin immaturity was evaluated in selected spermatozoa remaining after oocyte insemination by AB (n = 149) and CMA3 (n = 163) staining.

After sperm selection and fixation in paraformaldehyde [PFA, 500 µL, 4% in phosphate-buffered saline (PBS) pH 7.4, for 30 min at RT], 4 × 10^5^ spermatozoa were stained with 100 µL of CMA3 (Sigma Aldrich, St Louis, MO, USA) solution [0.25 mg/mL in McIlvane’s buffer (0.2 M Na_2_HPO_4_, 0.1 M citric acid), pH 7.0, containing 10 mM MgCl_2_], for 20 min at RT in the dark. Cells were then washed and resuspended in 10 µL of McIlvane’s buffer, pH 7.0, containing 10 mM MgCl_2_, smeared on slide, air-dried and mounted with PBS: glycerol (1:1). Two hundred spermatozoa were analyzed on each slide by fluorescence microscope (Axiolab A1 FL; Carl Zeiss, Milan, Italy), equipped with Filter set 49 and an oil immersion 100x magnification objective. Two types of staining patterns were identified: bright green fluorescence of the sperm head (abnormal chromatin packaging) and weak green staining (normal chromatin packaging) (Fig. 1A)^70^.

AB staining, which selectively stains lysine-rich histones^71^ was performed as previously described^70^. Briefly, after fixation in 4% PFA, 1 × 10^5^ spermatozoa were smeared on slide, air-dried and then stained with 5% aqueous AB (Sigma Aldrich, St Louis, MO, USA) mixed with 4% acetic acid (pH 3.5) for 5 min^72^ at RT. Two hundred spermatozoa were analyzed on each slide under a light microscope (Leica DM LS; Leica, Wetzlar, Germany). Spermatozoa showing dark-blue staining were considered as AB positive (Fig. 1B)^72^.

### Detection of CATSPER1

The extent of CATSPER1 expression in spermatozoa was determined in whole semen (n = 141) remaining after sperm preparation for ART, by an immunofluorescence-flow cytometric method, as previously described^23,24^. 10 × 10^6^ unselected spermatozoa were fixed in 4% PFA and washed twice in 1% NGS (normal goat serum, Sigma Aldrich, St Louis, MO, USA)-PBS, before permeabilization with 0.1% Triton X-100 in 100 µL 0.1% sodium citrate for 4 min in ice. After splitting into three identical aliquots, sperm samples were incubated for 1 hour at RT either with anti-CATSPER1 antibody (4 µg/ml, test sample, Santa Cruz Biotechnology, Dallas, TX, USA) or normal rabbit serum (4 µg/ml, Signet Laboratories, Hayward, CA, USA), the latter for negative control. The samples were washed twice in 1% NGS- PBS, and subsequently were incubated for 1 hour in the dark with goat anti-rabbit IgG-FITC (Southern Biotech, Birmingham, AL, USA) diluted 1:100 in 1% NGS-PBS. After two washing procedures, spermatozoa were resuspended in 300 µL PBS and incubated in the dark for 15 min at RT with 4.5 µL Propidium Iodide (PI, 50 µg/ml in PBS) to stain the nuclei. The third aliquot of spermatozoa was prepared with the same procedure but omitting the PI staining, for instrumental compensation. Samples were acquired using a flow cytometer (FACScan, Becton Dickinson, Mountain View, CA, USA) equipped with a 15-mW argon ion laser used at 488 nm for excitation. Green fluorescence of FITC-conjugated goat anti-mouse IgG was revealed by an FL-1 (515–555-nm wavelength band) detector; red fluorescence of PI was detected by an FL-2 (563–607-nm wavelength band) detector. We acquired 8000 nucleated events (i.e. the events stained with PI) in the gate of the characteristic forward scatter/side scatter region of sperm cells^73^. CATSPER1 expression in the different samples was expressed as median fluorescence intensity (MFI), calculated by the ratio between the median intensity of cells of the test sample and the median intensity of cells of the corresponding negative control (a fluorescence histogram depicting a negative control and a test sample is shown in Fig. 2A). Spermatozoa stained with the anti-CATSPER1 antibody used in our experiments were observed using Axiolab A1 FL (Carl Zeiss, Milan, Italy) fluorescence microscope using an oil immersion 100x magnification objective. The staining reveals a patchy and punctate pattern in the tail of most CATSPER positive spermatozoa (Fig. 2B)^23,24^.

### Statistical analysis

The following ART outcomes were considered: fertilization rate (FR, number of fertilized oocytes/number of inseminated oocytes); cleavage rate (CR, number of embryos/number of fertilized oocytes); good embryo quality (EQ_A_, number of embryos of A quality/number of total embryos); implantation rate (IR, number of gestational sac with fetal heart beat/number of transferred embryos); pregnancy rate (PR, number of clinical pregnancy/number of transferred embryos) and delivery rate (DR, number of delivery/number of clinical pregnancy).

Data were analyzed with SPSS (Statistical Package for the Social Sciences, Chicago, IL, USA), version 24.0 for Windows. Continuous variables that were found to be not normally distributed after the Kolmogorov-Smirnov test were expressed as median (interquartile range- IQR) value. Considering that no statistically significant differences were observed between IVF and ICSI for each outcome (not shown), the analysis was conducted in the entire cohort. Correlations were assessed using Spearman’s methods and Mann–Whitney U test was used for comparisons between groups. Multivariate analysis was performed to adjust data for confounding factors known to influence ART outcomes, such as female age^12,13^, female factors^14–16^ and number of MII oocytes^17^. For female factors only poor ovarian reserve, endometriosis and polycystic ovary syndrome were considered as, in ARTs, all other female factors are overcome by embryo transfer.

We used receiver operating characteristic (ROC) curve analysis to test the accuracy (as area under the curve, AUC) with 95% confidence interval, the sensitivity and the specificity, as well as to identify cut-off values of the different sperm variables (AB, CMA3 and CATSPER1) in predicting ART outcomes. Logistic regression was used to estimate adjusted odds ratios (OR) with 95% confidence intervals (CI).

Prediction models were constructed for those outcomes resulting correlated to one or more evaluated parameters. Predictors significantly associated with such outcomes were analyzed at multivariable logistic regression including in the model female age, female factor and number of MII oocytes as covariates. The performance of the models was quantified with respect to discrimination^74^, i.e. how the goodness of the model is able to distinguish between the two groups achieving or not the outcome, which was quantified with the ROC AUC. The reliability of the prediction produced by the model was statistically tested by the Hosmer-Lemeshow goodness-of-fit test.

All statistical tests were 2-sided, and P values of ≤0.05 were considered statistical significant.

### Data availability

The datasets generated during and/or analysed during the current study are available from the corresponding author on reasonable request.

## Electronic supplementary material

Supplementary Figure 1
