# Supplementary material for: Chromatin Protamination and Catsper Expression in Spermatozoa Predict Clinical Outcomes after Assisted Reproduction Programs
Source: Sci Rep. 2017 Nov 9;7:15122. doi: 10.1038/s41598-017-15351-3 (PMC5680250; doi:10.1038/s41598-017-15351-3)
Supplement: Supplementary file 1 — Supplementary Figure 1 [file 41598_2017_15351_MOESM1_ESM.pdf]

Supplementary Figure 1.

CHROMATIN PROTAMINATION AND CATSPER EXPRESSION IN SPERMATOZOA PREDICT CLINICAL OUTCOMES AFTER ASSISTED REPRODUCTION PROGRAMS

Marchiani S., Tamburrino L., Benini F., Fanfani L.,Dolce R., Rastrelli G., Maggi M., Pellegrini S., Baldi E.

A

CLEAVAGE (hours)	40/44	46/48	66/72	40/44	46/48	66/72	44/48	66/72	40/44	66/72
EMBRYO SCORE	B	B/C	E	A	B/C	E	A	D	B/C	A
	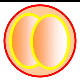 2 CELLS-STAGE			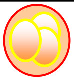 3 CELLS-STAGE			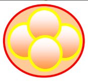 4 CELLS-STAGE		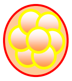 8 CELLS-STAGE	

B

PRESENCE OF MULTINUCLEATE BLASTOMERES	< 25%	> 25%
EMBRYO SCORE	D	E
	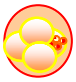	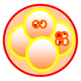

C

DEGREE OF FRAGMENTATION OF BLASTOMERES	< 10%	> 10%; < 30%	> 30%; < 50%	> 50%
EMBRYO SCORE	A	B	C	D
	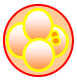	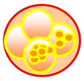	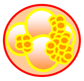	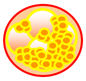

D

PRESENCE AND DEGREE OF ABNORMALITIES OF BLASTOMERES	Light dark, with low presence of granules and/or vacuoles	Dark, amorphous and with high presence of granules and vacuoles
EMBRYO SCORE	B/C-C	D-E
	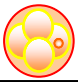	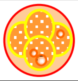

E

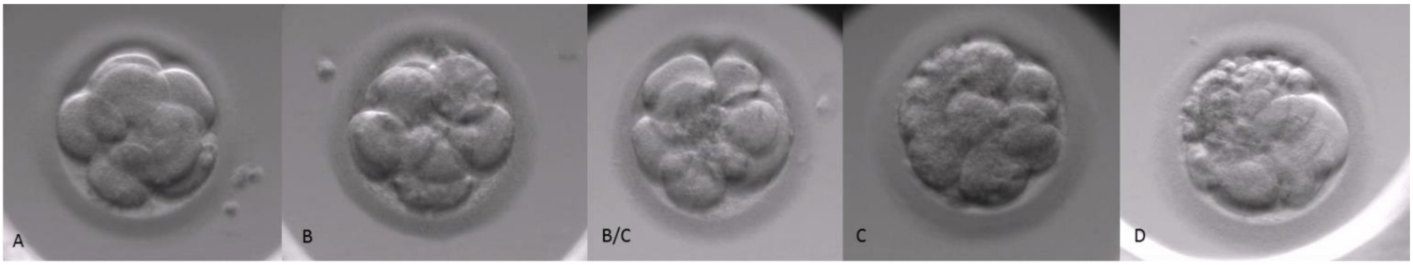

Supplementary Figure 1. Classification criteria of embryo quality. Criteria for embryo classification used in our study are: pace of division, degree of fragmentation and presence of abnormalities. For each criterion an embryo score (from A to E) was attributed. **a**: Schematic representation of cleavage time (hours from insemination). Score A was attributed when 2 cells-stage was observed at 40/44 hours or 4 cells-stage at 44/48 hours or 8 cells-stage at 66/72 hours. **b**: Schematic representation of different degree of blastomeres fragmentation. Score A was attributed when degree of fragmentation was less than 10%. **c**: Schematic representation of main blastomeres' abnormalities and their degree. Score A was attributed only in absence of any blastomeres' abnormalities. **d**: Images of 3-days embryos (obtained with Nikon Eclipse TE2000-S microscope using a 20x magnification objective) representing the different classes of quality, according to the above described criteria. In particular, Class A: excellent embryo quality; Class B: good embryo quality; B/C: fair embryo quality; C: sufficient embryo quality; D: quite sufficient embryo quality.
